# Supplementary material for: Psychological Disorders and Coping among Undergraduate College Students: Advocating for Students’ Counselling Services at Kuwait University
Source: Int J Environ Res Public Health. 2024 Feb 21;21(3):245. doi: 10.3390/ijerph21030245 (PMC10970382; doi:10.3390/ijerph21030245)
Supplement: Supplementary file 1 [file ijerph-21-00245-s001.zip › ijerph-2861545-supplementary.pdf]

Psychological disorders and coping among undergraduate college students: Advocating for students' counselling services at Kuwait University

Naser M. Alotaibi [1*], Moh A. Alkhamis [2], Mashael Alrasheedi [1], Khuloud Alotaibi [1], Latifa Alduaij [1], Fatmah Alazemi [1], Danah Alfaraj [1] and Danah Alrowaili [1]

Supplementary Table S1. Subscales and scoring of the Depression Anxiety and Stress Scale-21 (DASS-21) and Brief-COPE

Depression Anxiety and Stress Scale-21 (DASS-21)		
**Subscales**	**Scoring**	
	**No Psychological Symptoms (0)**<br>**Normal and Mild**	**Having Psychological Symptoms (1)**<br>**Moderate, Severe, and Extremely Severe**
**A) Depression**	0-13	≥ 14
**B) Anxiety**	0-9	≥ 10
**C) Stress**	0-18	≥ 19

Brief-COPE			
**Subscales**	**Scoring**		
	**Low Coping**<br>**(0-30%)**	**Medium Coping**<br>**(>30-70%)**	**High Coping**<br>**(>70%)**
**A) Problem-focused coping (0-32)**<br>1- Active coping<br>2- Use of informational support<br>3- Positive reframing<br>4- Planning	4 - 10	11 - 23	24 - 32
**B) Emotion-focused coping (0-48)**<br>1- Emotional support<br>2- Venting<br>3- Humour<br>4- Acceptance<br>5- Self-blame<br>6- Religion	6 - 15	16 - 34	35 - 48
**C) Avoidant coping (0-32)**<br>1- Self-distraction<br>2- Substance use<br>3- Denial<br>4- Behavioral disengagement	4 - 10	11 - 23	24 - 32

Supplementary Table S2. Univariate analysis of baseline characteristics without the study outcomes.

Characteristic	No Depression 461 (40.4%)	*p-value*	No Stress 554 (48.5%)	*p-value*	No Anxiety 351 (30.7%)	*p-value*
**Age**						
< 20 years old	238 (51.6)	< 0.01*	279 (50.4)	< 0.01*	324 (49.4)	< 0.01*
> 20 years old	223 (48.4)		275 (49.6)		332 (50.6)	
**Sex**						
Male	154 (33.4)	< 0.01*	182 (32.9)	< 0.01*	203 (31.0)	< 0.01*
Female	307 (66.6)		372 (67.1)		453 (69.1)	
**Marital Status**						
Single	382 (82.8)		467 (84.3)		556 (84.8)	
Married	68 (14.8)	0.08	73 (13.2)	0.50	83 (12.7)	0.80
Divorced	10 (2.2)		13 (2.4)		16 (2.4)	
Widowed	1 (0.2)		1 (0.12)		1 (0.2)	
**Socioeconomic Status**						
Low	42 (9.1)		54 (9.8)		68 (10.4)	
Medium	373 (80.9)	0.01*	447 (80.7)	0.01*	525 (80.0)	0.01*
High	46 (10.0)		53 (9.6)		63 (9.6)	
**Nationality**						
Citizen	391 (84.8)	< 0.01*	471 (85.0)	< 0.01*	539 (82.2)	0.01*
Resident	70 (15.2)		83 (15.0)		117 (17.8)	
**Faculty**						
Education	153 (33.2)		179 (32.3)		188 (28.7)	
Sharia and Islamic Studies	34 (7.4)		50 (9.0)		55 (8.4)	
Business Administration	64 (13.8)	< 0.01*	71 (12.8)	< 0.01*	75 (11.4)	< 0.01*
Science	55 (11.9)		71 (12.8)		80 (12.2)	
Allied Health	103 (22.3)		128 (23.1)		185 (28.2)	
Engineering and Petroleum	52 (11.3)		55 (9.9)		73 (11.1)	
**Year of Study**						
First	152 (33.0)		190 (34.3)		220 (33.5)	
Second	143 (31.0)	0.01*	158 (28.5)	< 0.01*	182 (27.7)	0.05
Third	77 (16.7)		97 (17.5)		110 (16.8)	
Fourth	89 (19.3)		109 (19.7)		144 (22.0)	
**Medical Illness**						
Yes	69 (15.9)	< 0.01*	86 (15.5)	< 0.01*	107 (16.3)	< 0.01*
No	392 (85.0)		468 (84.5)		549 (83.7)	
**Family Problems**						
Yes	45 (9.8)	< 0.01*	74 (13.4)	< 0.01*	107 (16.3)	< 0.01*
No	416 (90.2)		480 (86.6)		549 (83.7)	
**Coping Strategy**						
Problem Focused						
Low	68 (14.8)		71 (12.8)		75 (11.4)	
Moderate	314 (68.1)	< 0.01*	391 (70.6)	< 0.01*	450 (68.6)	< 0.01*
High	79 (17.1)		92 (16.6)		131 (20.0)	
Emotion Focused						
Low	147 (31.9)		156 (28.2)		165 (25.2)	
Moderate	311 (67.5)	< 0.01*	390 (70.4)	< 0.01*	482 (73.5)	< 0.01*
High	3 (0.70)		8 (1.4)		9 (1.4)	
Avoidant						
Low	81 (17.6)		87 (15.7)		93 (14.2)	
Moderate	295 (64.0)	< 0.01*	381 (68.8)	< 0.01*	440 (67.1)	0.04*
High	85 (18.4)		86 (15.5)		123 (18.8)	

* Statistically significant.
